# Renal perfusion improvement in the perioperative period after unilateral endovascular revascularization in patients with atherosclerotic renal artery stenosis

**DOI:** 10.3389/fcvm.2023.1193864

**Published:** 2023-07-12

**Authors:** Leyin Xu, Jiang Shao, Kang Li, Chaonan Wang, Zhichao Lai, Jiangyu Ma, Xiaoxi Yu, Fenghe Du, Junye Chen, Xiaolong Liu, Jinghui Yuan, Bao Liu, Chunyang Wang

**Affiliations:** ^1^ Department of Vascular Surgery, Peking Union Medical College Hospital (Dongdan Campus), Beijing, China; ^2^ Department of Urology, PLA General Hospital, Beijing, China

**Keywords:** renal artery stenosis, endovascular treatment, renal perfusion, flatpanel detector parenchymal blood volume (FD-PBV), atherosclerosis

## Abstract

**Background:**

The clinical benefits of endovascular treatment in renal artery stenosis (RAS) remain controversial. This study used an intraoperative renal perfusion imaging technique, called flat-panel detector parenchymal blood volume imaging (FD-PBV), to observe the change in renal perfusion after endovascular treatment in RAS.

**Materials and methods:**

In a prospective, single-center study, we assigned 30 patients with atherosclerotic RAS who underwent endovascular treatment between March 2016 and March 2021. The preoperative and postoperative results of renal perfusion, blood pressure, and renal function, were compared.

**Results:**

Both median kidney volume (*p* < 0.001) and median preoperative mean density of contrast medium (MDCM) (*p* = 0.028) increased significantly after endovascular treatment. The ratio of postoperative and preoperative MDCM differed greatly among the patients. For patients with preoperative MDCM <304.0 HU (Subgroup A, 15 cases), MDCM significantly increased after treatment (*p* = 0.001) and 12 (80.0%) patients had more than 10% increase in renal perfusion. For patients who had relatively high preoperative renal perfusion (MDCM ≥304.0 HU, Subgroup B, 15 cases), preoperative and postoperative MDCM were similar (*p* = 0.776). On the other hand, the serum creatinine levels significantly decreased in Subgroup A (*p* = 0.033) and fewer antihypertensive drugs were used after endovascular revascularization (*p* = 0.041). The preoperative and postoperative creatinine levels and number of antihypertensive drugs were similar in Subgroup B.

**Conclusions:**

During the perioperative period, RAS patients with relatively low preoperative renal perfusion levels had greater improvement in renal perfusion, renal function, and blood pressure control after endovascular treatment. The improvement of renal function needs to be confirmed by long-term follow-up.

## 1. Introduction

Endovascular treatment, including percutaneous transluminal renal angioplasty (PTRA) and renal artery stenting, is an important therapy for severe renal artery stenosis (RAS) (1, 2). However, the indications for endovascular treatment in atherosclerotic RAS are still under debate. Currently the assessment of RAS includes clinical parameters (renal function and blood pressure), and imaging techniques, such as computed tomography angiography (CTA), magnetic resonance angiography (MRA), Doppler ultrasonography, and digital subtraction angiography (DSA). Previous studies evaluated the predictive value of the renal resistive index (RRI) for the success of endovascular revascularization and had conflicting outcomes (3–6). Radermacher reported that patients with high RRI (>80) did not benefit from angioplasty or surgery (3). In patients with low-to-moderate RAS, low minimal luminal diameter was associated with low glomerular filtration rate (GFR), resistant hypertension, and cardiovascular events (7, 8). These imaging techniques assess the patency of renal arteries, but cannot show microvascular perfusion and the degree of renal parenchymal ischemia. The evaluation of RAS needs to be improved.

Renal perfusion is the blood flow at the level of the capillary bed, and is highly associated with the delivery of oxygen and nutrients (9). Renal perfusion is the key determinant of glomerular filtration. In kidney diseases, quantitative renal perfusion measurement can show the severity of renal parenchymal ischemia and directly reflect disease status (10). Thus, renal perfusion measurements can help assess the improvement in renal blood flow after renal artery stenting. A preliminary study showed that renal perfusion in patients with severe RAS was significantly lower than that in those with mild/modest RAS or healthy volunteers. In addition, renal perfusion differed greatly among patients with severe RAS (11). The correlation between preoperative renal perfusion and the clinical benefits of renal artery stenting remains unknown.

Our research group reported a new application named flat-panel detector parenchymal blood volume imaging (FD-PBV) in 2017, and it might be a new method for evaluating the perfusion of the target kidney during endovascular procedure (12). We designed the Perfusion of Renal Artery Disease Analysis (PRADA) clinical trial (NCT03252639, clinicaltrials.gov), and observed the change in renal perfusion after some RAS patients had renal artery stenting.

## 2. Materials and methods

### 2.1. Study design

This prospective, single-center study included 30 RAS patients who underwent renal artery stenting between March 2016 and March 2021 in the study hospital. The study complied with the Declaration of Helsinki (as revised in 2013) and the protocol was approved by the ethics committee and the institutional review board of the study hospital. We obtained written informed consent from each patient. The inclusion and exclusion criteria were as follows.

Inclusion criteria: (1) patients who provided informed consent; (2) patients who were diagnosed with RAS by CTA, MRA, or Doppler ultrasonography; (3) patients who were aged 18 years or older; (4) patients whose intervened renal arteries were not completely occluded (confirmed by DSA); (5) the stenosis percentage exceeding 70%; (6) unexplained recurrent congestive heart failure, sudden pulmonary oedema, uncontrolled blood pressure (>140/90 mmHg after at least 2 antihypertensive medications) or impaired renal function (GFR of the affected kidney <40 ml/min/1.73m^2^), and (7) patients whose affected kidney did not have total loss of function.

Exclusion criteria: patients who (1) were pregnant, (2) had nonatherosclerotic RAS, (3) had bilateral renal artery endovascular treatment, (4) had a history of renal transplantation or renal artery bypass surgery, (5) with multiple renal arteries of the affected side, (6) were allergic to iodine contrast medium, (7) had other contraindications of endovascular treatment, and (8) had technical failure in the endovascular treatment.

### 2.2. Outcomes

The mean density of contrast medium (MDCM), which reflected renal perfusion, was measured by FD-PBV. The ratio of MDCM was defined as postoperative MDCM divided by preoperative MDCM. Other endpoints included the change in blood pressure, number of antihypertensive drugs, serum creatinine levels, and kidney volumes. The GFR of the affected kidney was measured by ^99m^Tc-DTPA kidney dynamic imaging. Noninvasive blood pressure was measured at least twice a day (8 am and 4 pm) in the hospital, and the average pressure was calculated. Serum creatinine levels were tested in all patients before and the day after endovascular intervention. The kidney volumes were measured by FD-PBV.

### 2.3. Endovascular intervention

All endovascular interventions were performed by the same surgeon group. The detailed procedures are as follows. An introducer sheath was inserted into the femoral artery. Then, the 8F guiding catheter, 5F Cobra catheter, and guidewire were placed at the ostium of the target renal artery. DSA was used to evaluate the lesions. If the stenosis percentage was less than 70%, or the renal artery was completely occluded, the patients were excluded from the study. FD-PBV acquisition was run for the first time to analyze preoperative perfusion levels. All patients had either primary renal artery stenting, or PTRA plus stenting. DSA was performed a second time to confirm the patency of the arteries. Once again, we performed FD-PBV for postoperative results. The sheath was removed and a vascular closure device was used. A protocol for the reduction of contrast-induced nephropathy (normal saline 1 ml/kg/h from 12 h before to 24 h after the procedure) was used in patients with eGFR less than 60 ml/min/1.73m^2^.

### 2.4. FD-PBV acquisition

The dedicated FD-PBV acquisition protocol on a flat-panel detector angiographic system (Artis zeego; Siemens Healthcare, Forchheim, Germany) was employed based on two rotational runs, the mask run and fill run, with the following imaging parameters: acquisition time 8 s, tube voltage 70 kV, matrix 616 × 480, flat-panel detector size 30 cm × 40 cm, rotational angle 200°, 0.5°/frame, 400 frames in total, and dose 0.36 mGy/frame. After positioning of the catheter at the ostium of the renal artery, the mask run started before the contrast medium injection. Then, 20 ml diluted contrast medium (contrast medium 10 ml + saline 10 ml = 20 ml; contrast medium: Visipaque 320; GE Healthcare, Milwaukee, WI) was injected at a rate of 4 ml/s through the catheter. Later, the fill run was triggered manually when opacification of the kidney cortex was seen, marking the steady state of contrast medium filling for renal parenchyma. To reduce motion artifacts, patients were instructed to hold their breath during PBV acquisition. The acquisition of all patients was performed by the same engineer from Siemens AG.

### 2.5. Data analysis

The system automatically transferred the acquired data to the workstation (syngo X-Workplace; Siemens Healthcare GmbH, Forchheim, Germany) for postprocessing using commercially available imaging software (syngo Neuro PBV; Siemens AG Healthcare Sector, Germany). PBV reconstruction was visualized in the form of colored multiplanar reconstruction images, in which the pseudocolor corresponded to blood volume. The kidney volume and the mean density of CM of the whole kidney were measured using the syngo volume task card tool. The data of all patients were analyzed by the same engineer from Siemens AG.

### 2.6. Statistical analysis

Kidney volumes, MDCM and serum creatinine levels are presented as the median with interquartile range, and blood pressure, and antihypertensive drugs are presented as the mean ± standard deviation. The preoperative and postoperative results were compared by the Wilcoxon signed rank test, or paired Student’s t test. The correlations between the ratio of MDCM and other factors were analyzed by Pearson correlation analysis. In addition, stepwise multifactor linear regression model was used. The patients were divided into two subgroups depending on the preoperative MDCM. The cutoff value was the median of MDCM. Subgroup A included patients with relatively low preoperative MDCM, and Subgroup B included patients with relatively high preoperative MDCM. The kidney volumes, MDCM, serum creatinine levels, blood pressure, and antihypertensive drugs of different subgroups were compared by the Wilcoxon rank sum test or Student’s t test. A *p*-value less than 0.05 was considered statistically significant. A specialist in medical statistics participated in all statistical analyses. All analyses were conducted using IBM SPSS Statistics software for Windows, version 22.0 (Armonk, NY, USA).

## 3. Results

Thirty patients were included in the study and the baseline characteristics are summarized in Table 1. The mean age of all patients was 59.8 ± 9.5 years, and 22 (73.3%) were men. Comorbidities included hypertension (30/30, 100%), diabetes mellitus (4/30, 13.3%), cerebrovascular disease (6/30, 20.0%), coronary artery disease (5/30, 16.7%), and other peripheral vascular diseases (19/30, 63.3%). Twenty-one (70.0%) of the intervened renal arteries had more than 90% stenosis. Fifteen (50.0%) patients underwent balloon angioplasty plus stenting, and the other 15 (50.0%) patients had primary renal artery stenting.

**Table 1:** Baseline characteristics of patients with renal artery stenosis.

	Total (*N* = 30)	Group A (*N* = 15)	Group B (*N* = 15)	*p* value
Age, years	59.8 ± 9.5	59.7 ± 6.3	59.9 ± 12.2	0.955
Sex, male, *n*(%)	22 (73.3)	10 (66.7)	12 (80.0)	0.682
Comorbidities, *n*(%)				
Hypertension	30 (100)	15 (100)	15 (100)	1.000
Diabetes mellitus	4 (13.3)	4 (26.7)	0 (0)	0.100
Cerebrovascular diseases	6 (20.0)	6 (40.0)	0 (0)	0.017
Coronary artery diseases	5 (16.7)	0 (0)	5 (33.3)	0.042
Other peripheral artery diseases	19 (63.3)	13 (86.7)	9 (60.0)	0.215
Antihypertensive drugs, *n*(%)				
Calcium channel blockers	22 (73.3)	11 (73.3)	11 (73.3)	1.000
Angiotensin receptor blockers	12 (40.0)	5 (33.3)	7 (45.7)	0.710
Beta-receptor blockers	7 (23.3)	3 (20.0)	4 (26.7)	1.000
Alpha-receptor blockers	6 (20.0)	3 (20.0)	3 (20.0)	1.000
Diuretics	6 (20.0)	3 (20.0)	3 (20.0)	1.000
Affected kidney, *n*(%)				
Left	16 (53.3)	11 (73.3)	5 (33.3)	0.066
Right	14 (46.7)	4 (26.7)	10 (66.7)	
Renal artery diameter, mm	8.4 ± 1.9	8.9 ± 2.3	7.9 ± 1.2	0.139
Minimal luminal diameter, mm	1.0 ± 0.7	1.0 ± 0.7	1.0 ± 0.6	0.935
Stenosis percentage, *n*(%)				
70%–90%	9 (30.0)	3 (20.0)	6 (40.0)	0.427
>=90%	21 (70.0)	12 (80.0)	9 (60.0)	
GFR of affected kidney, ml/min/1.73m^2^	29.6 (18.3–36.6)	28.9 (14.8–36.7)	29.6 (20.9–37.8)	0.694

### 3.1. Renal function and blood pressure

Table 2 shows the comparison of preoperative and postoperative renal function and blood pressure. The preoperative and postoperative creatinine levels were similar (*p* = 0.166), indicating that FD-PBV was safe for RAS patients. Compared with their baseline creatinine levels, only two patients had more than a 10 μmol/L increase (patient 1: 71 μmol/L→90 μmol/L; patient 2: 104 μmol/L→124 μmol/L). The creatinine levels of these two patients decreased to baseline in a week.

**Table 2:** Preoperative and postoperative results in patients who underwent endovascular revascularization.

	Pre-operation	Post-operation	*p* value
Serum creatinine, μmol/L	88 (79, 106)	90 (74, 108)	0.166
Number of antihypertensive drugs	1.7 ± 0.9	1.5 ± 1.0	0.012
Mean systolic blood pressure, mmHg	136.7 ± 16.0	128.1 ± 11.1	0.001
Mean diastolic blood pressure, mmHg	79.2 ± 11.2	74.7 ± 10.6	0.007
Kidney volume, cm^3^	141.5 (106.5, 181.7)	148.6 (129.6, 186.3)	<0.001
Mean density of contrast medium, HU	304.0 (224.8, 380.9)	339.9 (283.8, 396.8)	0.028

Data are presented as the mean ± standard deviation, or median with interquartile range. HU = Hounsfield unit.

The mean preoperative number of antihypertensive drugs was 1.7 ± 0.9 and the mean postoperative number of antihypertensive drugs was 1.5 ± 1.0. The patients used fewer antihypertensive drugs during the perioperative period (*p* = 0.012). On the other hand, both systolic and diastolic blood pressure significantly decreased. The mean systolic blood pressure was 136.7 ± 16.0 mmHg before the operation and 128.1 ± 11.1 mmHg after the operation (*p* = 0.001). The mean diastolic blood pressure was 79.2 ± 11.2 mmHg before the operation and 74.7 ± 10.6 mmHg after the operation (*p* = 0.007).

### 3.2. Renal perfusion

**Figure 1:**
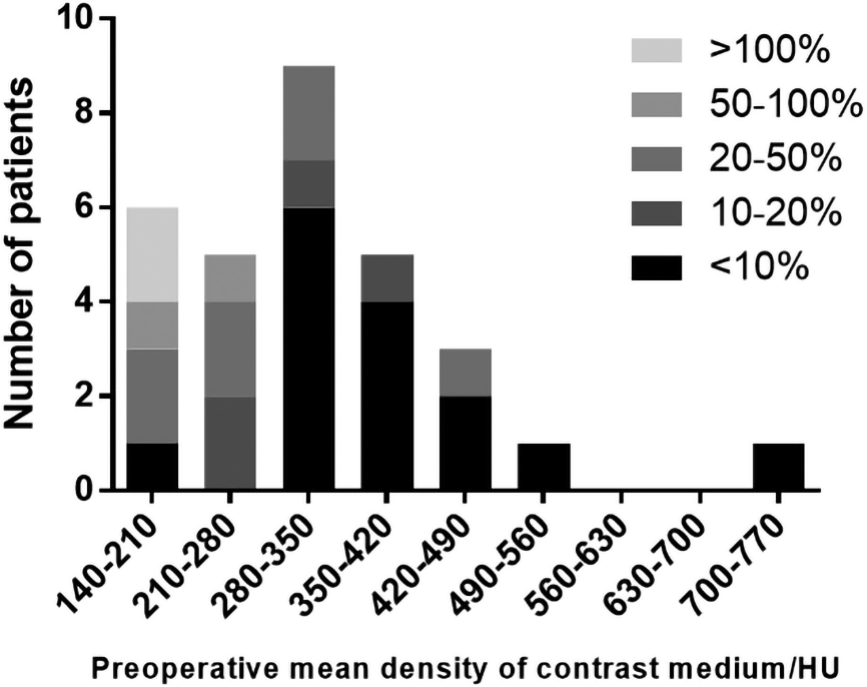
The ratio of the mean density of contrast medium in patients with different preoperative perfusion levels.

**Figure 2:**
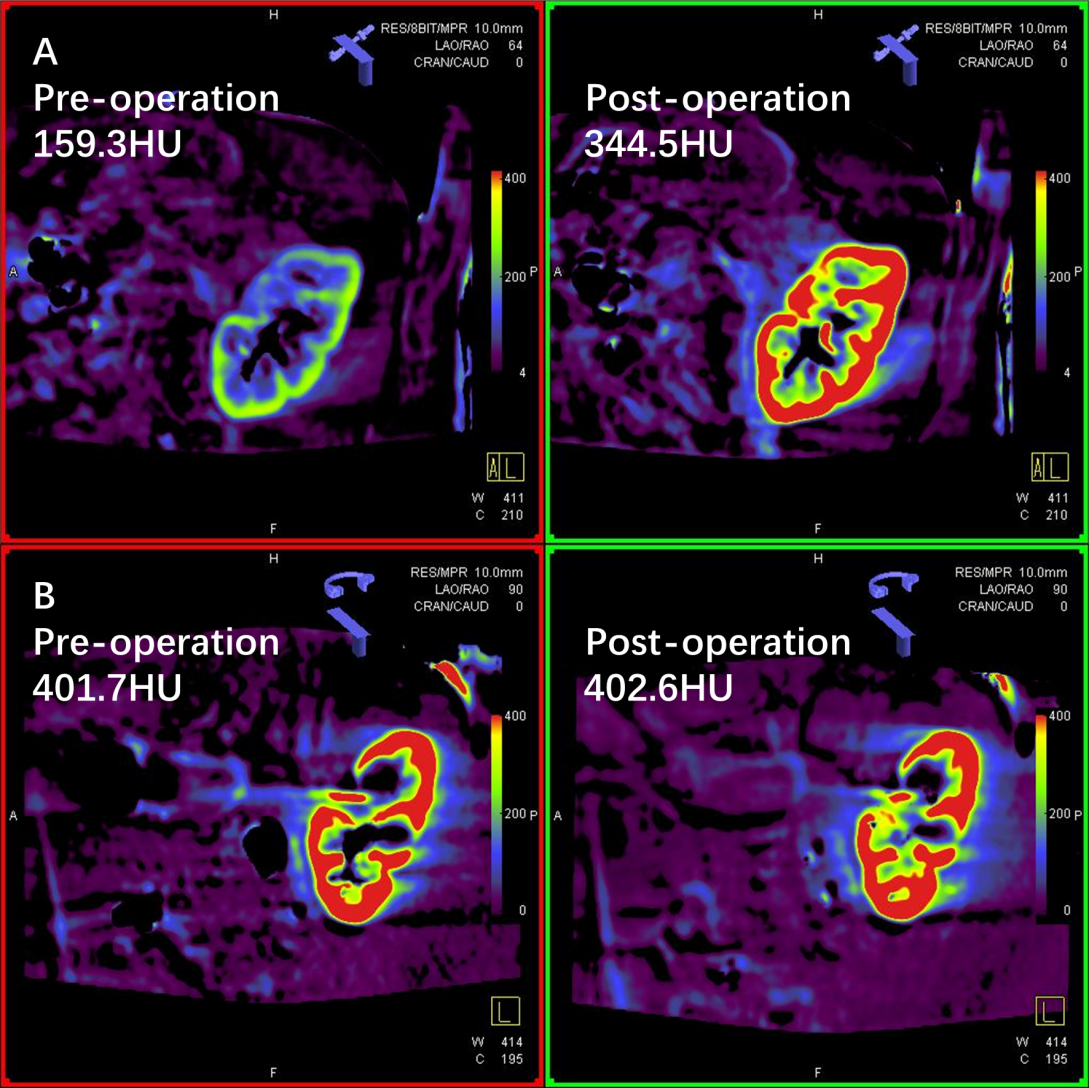
Two cases of renal perfusion change after unilateral revascularization. (**A**) Flat-panel detector parenchymal blood volume imaging (FD-PBV) of a patient whose renal perfusion significantly improved after endovascular revascularization (mean density of contrast medium 159.3 HU→344.5 HU); (**B**) FD-PBV imaging of a patient who had no renal perfusion improvement after endovascular revascularization (mean density of contrast medium 401.7 HU→402.6 HU).

We compared the preoperative and postoperative results acquired from the FD-PBV (Table 2). The median kidney volume increased significantly after revascularization (from 141.5 to 148.6 cm^3^, *p* < 0.001). The median MDCM was 304.0 HU before revascularization, and it increased to 339.9HU after revascularization (*p* = 0.028). However, not all patients had significant improvement in MDCM. The ratio of MDCM was distributed as follows (Figure 1): less than 10% increase (15 patients), 10%–15% increase (11 patients), 50%–100% increase (2 patients), and more than 100% increase (2 patients). Figure 1 shows one patient who had significant improvement in renal perfusion (Figure 2A) and one patient who had no change (Figure 2B). In the patients with a stenosis percentage <90%, the mean value of the natural logarithm of the ratio of MDCM was −0.096 ± 0.210 and was significantly lower than that in the patients with a stenosis percentage >=90% (0.218 ± 0.304, *p* = 0.009).

#### 3.1.1. Correlation analysis

The correlations between the ratio of MDCM and other influencing factors are listed in Table 3. Preoperative MDCM and the ratio of MDCM were negatively correlated (*R* = 0.698, *p* < 0.001). On the other hand, stenosis percentage and the ratio of MDCM were negatively correlated (*R* = 0.420, *p* = 0.021). In the stepwise multifactor linear regression model for predicting the natural logarithm of the ratio of MDCM, only preoperative MDCM was included (constant = 0.687, B = −0.002, *R* = 0.698, *p* < 0.001).

**Table 3:** Correlation analysis of natural logarithm of the ratio of mean density of contrast medium.

	Pearson correlation	*p* value
Age	−0.130	0.494
GFR of affected kidney	−0.203	0.290
Number of antihypertensive drugs	0.042	0.827
Renal artery diameter	0.301	0.105
Minimal luminal diameter	−0.288	0.123
Stenosis percentage	0.420	0.021
Preoperative kidney volume	−0.149	0.431
Preoperative mean density of contrast medium	−0.698	<0.001

### 3.2. Subgroup analysis

We used the median of preoperative MDCM (304.0 HU) as the cutoff value. Both Subgroups A and B included 15 patients. The stenosis percentage was similar between the two subgroups (Table 1). Twelve patients (80.0%) in Subgroup A and 3 patients (20.0%) in Subgroup B had a more than 10% increase in MDCM, and the ratio was significantly different between the two subgroups (*p* = 0.003). The comparisons of kidney volumes, renal perfusion, blood pressure, and renal function between the two subgroups are shown in Table 4. The kidney volumes of both subgroups increased significantly after intervention. The MDCM of Subgroup A was increased significantly (*p* = 0.001), but there was no significant change in Subgroup B (*p* = 0.776). On the other hand, the renal function of Subgroup A showed significant improvement (*p* = 0.033), but preoperative and postoperative serum creatinine levels were similar in Subgroup B (*p* = 0.937). The blood pressure of both subgroups decreased significantly after intervention. The postoperative number of antihypertensive drugs in Subgroup A was significantly lower than that before surgery (*p* = 0.041). The mean preoperative and postoperative numbers of antihypertensive drugs in Subgroup B were not significantly different (*p* = 0.164).

**Table 4:** Changes in renal function, blood pressure, and renal perfusion in two subgroups.

	Subgroup A				Subgroup B				*p* value^‡^
	Pre-operation	Post-operation	Difference	*p* value^†^	Pre-operation	Post-operation	Difference	*p* value^†^	
Serum creatinine, μmol/L	100 (79, 130)	99 (73, 118)	−6 (−11, −1)	0.033	84 (77, 97)	85 (78, 99)	−1 (−4.8, 7.5)	0.937	0.151
Number of antihypertensive drugs	1.7 ± 1.0	1.4 ± 1.0	−0.27 ± 0.46	0.041	1.8 ± 0.8	1.7 ± 1.0	−0.13 ± 0.35	0.164	0.379
Mean systolic blood pressure, mmHg	136.1 ± 13.9	129.5 ± 8.8	−6.6 ± 10.7	0.032	137.2 ± 18.4	126.6 ± 13.2	−10.6 ± 15.8	0.021	0.421
Mean diastolic blood pressure, mmHg	78.2 ± 10.0	74.9 ± 9.9	−3.3 ± 10.3	0.233	80.1 ± 12.5	74.5 ± 11.6	−5.6 ± 6.2	0.003	0.465
Kidney volume, cm^3^	144.7 (101.4, 183.3)	152.5 (119.1, 186.3)	7.8 (5.0, 17.8)	0.012	138.4 (119.4, 181.4)	148.4 (132.8, 186.3)	10 (−1.2, 16)	0.006	0.870
Mean density of contrast medium, HU	225.7 (183.3, 284.6)	297.6 (267.1, 351.1)	62.5 (28.5, 129.6)	0.001	373.9 (341.2, 457.0)	369.5 (325.9, 451.1)	−7.2 (−113, 26)	0.776	0.001

Data are presented as the mean ± standard deviation, or median with interquartile range. HU, Hounsfield unit. Difference, post-operation value—pre-operation value.

^†^ Wilcoxon signed rank test or paired student t test to compare the preoperative and postoperative results in the subgroup.

^‡^ Wilcoxon test or student t test to compare the difference values between subgroup A and B.

## 4. Discussion

Renal artery stenosis is one of the common causes of secondary hypertension and it may result in chronic renal dysfunction (2, 13). Endovascular treatment has become the main method for patients who need revascularization because of its minimal invasiveness (14). However, compared with medical therapy, previous studies have not shown significant clinical benefits of endovascular revascularization (15–17). These trials had some limitations. The severity of renal artery stenosis in the STAR trial was defined by noninvasive examinations (15), and 18.8% of the patients in the stenting group did not have any endovascular treatment because the stenosis percentages were less than 50% by DSA. In addition, the study only included patients with well-controlled blood pressure. Approximately 40% of the patients in the ASTRAL trial had less severe stenosis (<70%) (16). On the other hand, approximately one-third of the patients in the CORAL trial were diabetic, which meant that the renal failure in some patients might not be due to RAS (17). The inclusion criteria for these trials might be overly liberal and dilute the potential benefit of endovascular treatment (18). Current methods cannot help clinicians select RAS patients who may benefit from endovascular treatment.

Current diagnostic imaging techniques include CTA, MRA, Doppler ultrasonography, and DSA (19). These methods can assess the patency of the renal artery. However, none of them can quantitatively assess the microvascular perfusion of the kidney. In kidney diseases, renal perfusion measurements can show the severity of renal parenchymal ischemia and directly reflect disease status (10). Perfusion imaging techniques are rarely used in clinical practice (20). Computed tomography (CT) can be used to assess whole-organ perfusion, but the use of a large amount of contrast medium may lead to kidney damage (21). Only a small number of studies have investigated the renal perfusion changes after revascularization and had conflicting results. Koivuviita reported in 2012 that renal perfusion measured by Positron emission tomography (PET) did not change after revascularization (22). Mahmud evaluated renal perfusion of RAS patients and suggested that patients with great perfusion improvement after revascularization had better control of blood pressure in the follow-up (23).

FD-PBV was first used in the cerebral perfusion analysis of patients with cerebrovascular diseases. A pilot study compared the cerebral blood volumes measured by FD-PBV and perfusion CT in patients with acute symptoms of cerebral ischemia, and showed good correlation of these two techniques (correlation coefficient 0.72; *p* < 0.001) (24). Another study by Struffert also confirmed the accuracy of FD-PBV to measure cerebral blood volumes in acute middle cerebral artery occlusion patients (25). In liver tumors, the blood volumes measured by PBV and perfusion CT also showed a significant correlation (*r* = 0.97, *p* < 0.01) (26). The accuracy of FD-PBV has been validated by these studies. In addition, a study of liver perfusion in a swine model demonstrated that FD-PBV was highly reproducible (27).

This perfusion imaging technique has been used in some organs (such as the brain, liver, lung, and muscles) and shows favorable outcomes (25, 28–31). Our research team first introduced the applications of FD-PBV to renal perfusion analysis (12). This technique can quantitatively evaluate renal perfusion without moving patients from the operation table, which enables surgeons to obtain the real-time perfusion levels during the operation. To minimize the variation of manual triggering, the whole image acquisition process was operated by the same engineer from Siemens, who is proficient in PBV acquisition. Although we used extra contrast medium for FD-PBV acquisition, the amount of contrast medium was acceptable (10 ml for each acquisition). Only two of the patients had a transient increase in creatinine after endovascular intervention. FD-PBV is safe and effective for renal perfusion evaluation in patients who undergo endovascular revascularization.

In this study, the volumes of the intervened kidneys increased significantly after endovascular treatment due to the improvement in the patency of renal arteries. However, the change in renal perfusion varied in different patients. The change in MDCM was highly associated with preoperative MDCM, and patients with relatively low preoperative perfusion levels were more likely to have perfusion improvement. The subgroup analysis showed that renal perfusion after surgery in Subgroup A was significantly higher than before surgery. A total of 80.0% (12/15) of Subgroup A patients had a more than 10% perfusion increase. However, only 20.0% (3/15) of Subgroup B patients had a more than 10% perfusion increase. On the other hand, the changes in blood pressure and renal function were different between the two subgroups. The renal functions of Subgroup A patients improved after revascularization and they used fewer antihypertensive drugs. These changes were in accordance with the change in renal perfusion. A previous study demonstrated that low renal perfusion was associated with more blood pressure reduction after renal artery stenting in hypertensive patients (32). Patients who had relatively low initial renal perfusion may be more likely to have perfusion improvement during the perioperative period.

Because of its invasive nature, FD-PBV can only be utilized in RAS patients who are inclined to endovascular intervention. We did not have renal perfusion results of patients with mild or modest renal artery stenosis, or of normal people as a reference. Therefore, noninvasive, quantitative perfusion imaging techniques could be a feasible approach for preoperative assessment. Potential noninvasive perfusion imaging techniques include dynamic contrast-enhanced magnetic resonance imaging, arterial spin labeling, and contrast-enhanced ultrasound, but the clinical application of these perfusion imaging methods needs to be further studied (11, 33, 34).

The study had the following limitations. First, to minimize the potential risks of renal damage, the FD-PBV results were not repetitively measured. Second, as the size of the study was relatively small, the strength of the evidence needs to be further improved. Third, the change in renal perfusion during follow-up could not be evaluated. Fourth, the creatinine levels of 48 h and 72 h after the procedure were not measured in most patients.

In conclusion, during perioperative period, the perfusion improvement of RAS patients was negatively correlated with the preoperative perfusion levels. Patients who had relatively low initial perfusion levels tended to have more improvement in renal perfusion, renal function, and blood pressure reduction. The improvement of renal perfusion after endovascular revascularization needs to be confirmed by long-term follow-up.

## Data Availability

The raw data supporting the conclusions of this article will be made available by the authors, without undue reservation.
